# Bird conservation would complement landslide prevention in the Central Andes of Colombia

**DOI:** 10.7717/peerj.779

**Published:** 2015-02-19

**Authors:** Natalia Ocampo-Peñuela, Stuart L. Pimm

**Affiliations:** Nicholas School of the Environment, Duke University, Durham, NC, USA

**Keywords:** Endemic species, Forest restoration, Conservation policy, Landslide prevention, Ecosystem services, Manizales

## Abstract

Conservation and restoration priorities often focus on separate ecosystem problems. Inspired by the November 11th (2011) landslide event near Manizales, and the current poor results of Colombia’s Article 111 of Law 99 of 1993 as a conservation measure in this country, we set out to prioritize conservation and restoration areas where landslide prevention would complement bird conservation in the Central Andes. This area is one of the most biodiverse places on Earth, but also one of the most threatened. Using the case of the Rio Blanco Reserve, near Manizales, we identified areas for conservation where endemic and small-range bird diversity was high, and where landslide risk was also high. We further prioritized restoration areas by overlapping these conservation priorities with a forest cover map. Restoring forests in bare areas of high landslide risk and important bird diversity yields benefits for both biodiversity and people. We developed a simple landslide susceptibility model using slope, forest cover, aspect, and stream proximity. Using publicly available bird range maps, refined by elevation, we mapped concentrations of endemic and small-range bird species. We identified 1.54 km^2^ of potential restoration areas in the Rio Blanco Reserve, and 886 km^2^ in the Central Andes region. By prioritizing these areas, we facilitate the application of Article 111 which requires local and regional governments to invest in land purchases for the conservation of watersheds.

## Introduction

From October 19th to 29th, 2011, an estimated 400,000 people in the city of Manizales, in the Central Andes of Colombia, lost all access to their water supply. The cause was a major landslide that broke two main pipes transporting water from the cloud forests and paramos to the city, causing millions of dollars of economic loss.

In montane areas with abundant rainfall, seismic activity, volcano-ice interactions and natural erosion processes, landslides are a natural process and key disturbances (Huggel et al., 2007; Restrepo & Alvarez, 2006). The overexploitation of natural resources and deforestation increase the magnitude and frequency of landslides by removing the vegetation and root matrix that hold soil in place, however (Allan, 2004; Keefer & Larsen, 2007). Climate change will likely worsen the frequency and magnitude of landsides (Nadim et al., 2006). Cleared forests on steep slopes are especially vulnerable to landslides, while areas affected by landslides are ideal for restoration of forest cover. Restoring forests would reduce the risk of further disasters.

Tropical montane areas also house high levels of biodiversity. The northern Andes have high numbers of endemic and threatened vertebrate taxa (Jenkins, Pimm & Joppa, 2013). Similarly, Joppa, Roberts & Pimm (2010) showed the region to have exceptional numbers of plant species, many of which are endemic, and they predicted it to have large numbers of undiscovered species. In particular, the Central Andes holds 56 endemic and small-ranged bird species (Ocampo-Peñuela & Pimm, 2014). Human settlement and land clearing for agriculture (Etter et al., 2006; Etter et al., 2011) have extensively fragmented their ranges (Ocampo-Peñuela & Pimm, 2014), so many species are at risk of extinction.  Forest restoration in areas of high endemism and small-ranged bird species would have the greatest conservation benefit as exemplified also by Important Bird Areas (Franco et al., 2009).

We prioritize conservation and restoration areas in the Central Andes of Colombia, with a localized case study in the Rio Blanco Reserve, near the city of Manizales. Our aim is to find places where conserving or restoring an area would provide habitat for endemic and threatened bird species, while contributing to landslide prevention. We then extrapolate this exercise to the entire Central Andes ecoregion where comparable landslide occurrence data were not available.

Colombia’a Article 111 of Law 99 of 1993 provides for the purchase of land for conservation and restoration in watersheds that provide water to towns and cities. We conducted this priority setting exercise to facilitate the implementation of Article 111 at the Rio Blanco Reserve (Aguas de Manizales), and at other municipalities in Colombia. Article 111 states that “all municipalities and departments must invest at least 1% of their revenue in purchasing or maintaining land that protects watersheds, or in paying for ecosystem services that contribute to the same goal during 15 years, starting in 1999” (Ministerio del Medio Ambiente, 1993). Although this Article provides an avenue for the conservation of biodiverse lands, it has rarely been implemented.

Local and regional governments have largely neglected the law. After 15 years, only 0.12% of the revenue was invested in land purchasing and fewer than half of the municipalities, and a third of the departments implemented the law (Rudas, 2010). In some cases, the revenue funds have been spent on other activities. In other cases, local governments have found it difficult to identify the land to purchase for conservation (Rudas, 2010). In response, Decree 0953 delineated new guidelines for Article 111 and was published in 2013 (Ministerio de Ambiente y Desarrollo Sostenible, 2013). The new decree details the source of the 1% of the revenue and lays out specific investment rules. The new decree allows local governments to pay for ecosystem services, such as hydrological regulation and sediment and erosion control, as they ready for land purchases.

Prioritization strategies are highlighted to include: improvement of water quality, presence of water sources, aquifer conservation, natural land cover, areas vulnerable to anthropogenic pressure, and ecosystem connectivity (Ministerio de Ambiente y Desarrollo Sostenible, 2013). The goal of our study was to contribute to developing these strategies for identifying lands that should be purchased for conservation. We do this by mapping areas important for conservation of existing forest, and restoration to natural forest of current cattle pasture and croplands.

## Materials and Methods

### Study area

Bird diversity in Colombia is the world’s highest with 1834 species, with 77 endemic species (Stiles et al., 2014). Of the total, 106 species are listed as threatened by the International Union for Conservation of Nature (IUCN) (Bird Life International, 2014). The high diversity is partly explained by the unique geography of the Andes in Southwestern Colombia, where it divides into three cordilleras: Eastern, Western, and Central. All three are identified as priority areas for biodiversity (Jenkins, Pimm & Joppa, 2013) and are inside the Tropical Andes biodiversity hotspot (Myers et al., 2000), one of the three hotspots found in Colombia. The Western Andes hold the highest numbers of endemic and small-range bird species, followed by the Eastern cordillera (Cuervo et al., 2003; Kattan et al., 2004; Ocampo-Peñuela & Pimm, 2014). Although the Central Andes are not as diverse as the other two cordilleras (Kattan et al., 2004), they house 16 endemic and 6 threatened species in a given place (Ocampo-Peñuela & Pimm, 2014). Importantly, the Central Andes is the most threatened cordillera: over 70% of Colombia’s population has settled on the Andes with highest densities in the Central Andes (Etter & Van Wyngaarden, 2000), resulting in significant land conversion for agriculture (Etter et al., 2011), and habitat fragmentation (Etter et al., 2006).

Coupled with high bird diversity, the Andes are prone to landslides and avalanches due to their steep terrain and high rainfall (Huggel et al., 2010; Nadim et al., 2006). The Central Andes are specifically vulnerable to these phenomena due to their characteristic geological and geomorphic conditions, wet climate, and location. Our specific study area is located near the Ruiz and Bravo volcanoes, on a seismically active region, increasing its vulnerability to erosion and landslides locally (Westen & Terlien, 1996).

**Figure 1:**
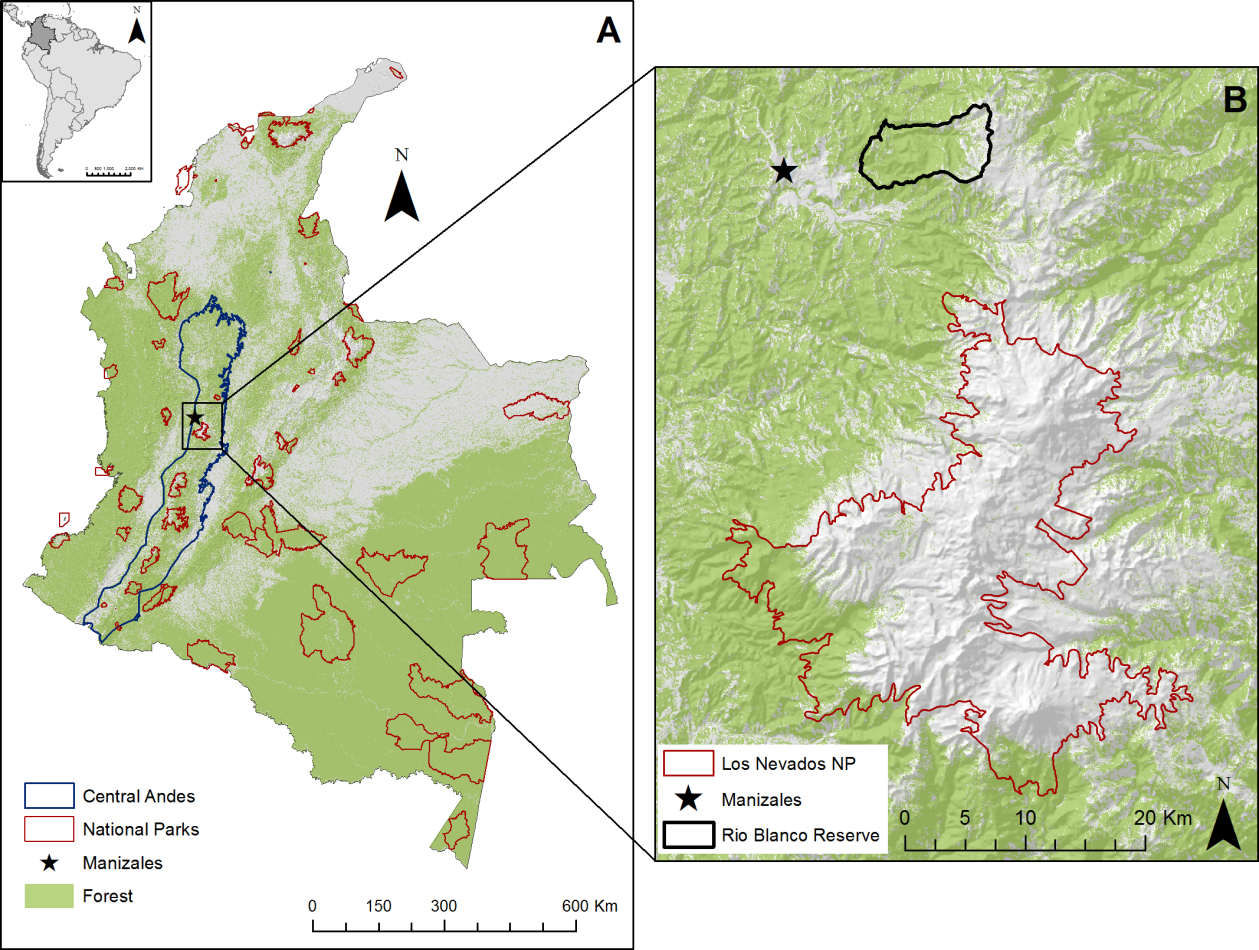
Location of study area. (A) Map of Colombia showing forest cover, National Parks, and the Central Andes region. (B) Localized study area near Manizales, Rio Blanco Reserve and the Los Nevados National Park.

We studied the Reserva Forestal Protectora de Río Blanco y Quebrada Olivares (hereafter the Rio Blanco Reserve), located 3.5 km from the city of Manizales, on the western slope of the Central Andes of Colombia, in the department of Caldas (Fig. 1). The Rio Blanco Reserve covers 49.32 km^2^, with 17.76 km^2^ (36%) in pasture for cattle and 0.8 km^2^ in pasture for cattle rotating with potato crops; the rest is forest (62%). The elevation rises from 2,000 to 3,800 m above sea level and the dominant ecosystem is cloud forest. It has some paramo areas at higher elevations and houses 409 species of plants, 344 birds, and 41 mammals (Corpocaldas et al., 2010).

Rio Blanco Reserve was declared a protected area in 1990 as part of the conservation corridor around Los Nevados National Park, which conserves 49 km^2^ of paramo, Andean forests, and glaciers (Fig. 1B). The reserve currently provides 35% of the potable water to the city of Manizales. Some of the threats to the forests of the area include landslides, cattle ranching, potato plantations, and densely planted introduced trees (Corpocaldas et al., 2010).

### Landslide susceptibility index

A landslide susceptibility index expresses the likelihood of landslide occurrence in an area based on local terrain conditions (Brabb, 1984). We developed a simple landslide susceptibility index for the Rio Blanco Reserve using slope, aspect, stream proximity, and the presence or absence of forest cover. Other studies have developed susceptibility indices using a suite of variables, including rainfall (Farahmand & AghaKouchak, 2013), slope, aspect, stream proximity, and landcover (Perotto-Baldiviezo et al., 2004). Landcover, slope and aspect tend to be the strongest predictors of landslide hazard (Perotto-Baldiviezo et al., 2004).

Our landslide susceptibility index ranges from 0 to 100, with 100 indicating the highest risk. All input variables were transformed into weighted index values following guidelines used by Perotto-Baldiviezo et al. (2004), but adapting the weights to the local context. The variable with the highest weight was slope (40), followed by forest cover (30), stream proximity (20), and aspect (10) (Table 1).

**Table 1:** Input variables to the landslide susceptibility index, and their associated weighted values, for the Rio Blanco Reserve.

Layer	Categories	Index value
**Slope**	0–12%	0
	12–30%	10
	30–60%	20
	60–100%	30
	>100%	40
**Forest cover**	Forest	10
	No forest (grass, crops, bare soil)	30
**Stream proximity**	0–300 m	20
	300–500 m	10
	>500 m	0
**Aspect**	N	0
	E	5
	W	5
	S	10

We derived the slope and aspect from a 90 m Digital Elevation Index (Jarvis et al., 2008) using tools from ArcMap 10.2 (ESRI, 1999–2010) and downscaling the resolution to 30 m using nearest neighbor cell resampling. We generated a slope scale of 0–100% steepness, and divided the aspect into 4 directions (Table 1). We derived the forest cover (forest/not forest) from data produced by Hansen et al. (2013) at a 30 m resolution for the world using LANDSAT images. Stream proximity was calculated in three categories using the Kernel Density tool in ArcMap 10.2 (ESRI, 1999–2013). The resulting landslide susceptibility index has a 30 m resolution.

We tested the accuracy of the landslide susceptibility index using georeferenced data collected in 2013 for landslide occurrence. We used 68 points for landslide presence and 29 for landslide absence. Aguas de Manizales personnel collected these points both inside the Rio Blanco Reserve and in areas 10 km to the south of it. In January of 2014, we collected data in different types of landcover (forest, open grass, elfin forest, and paramo), where landslides had not occurred in order to test our landslide susceptibility index. We used a confusion matrix to determine the overall accuracy of our landslide susceptibility index as well as user’s and producer’s accuracy (also known as commission and omission error). This is the most widely used method in remote sensing (Foody, 2002). We could not test the index’s accuracy more widely in the Central Andes regions due to the lack of publically available georeferenced landslide data.

### Birds

For the purpose of this study, we considered only terrestrial diurnal bird species that were either endemic or had a range smaller than 100,000 km^2^, and whose range included the Central Andes region. We started with the range maps provided by Bird Life International and NatureServe (2013) and refined it by suitable elevation following each species’ altitudinal requirements. (The detailed methods are in Harris, Jenkins & Pimm (2005) and Ocampo-Peñuela & Pimm (2014)). The resulting range included only areas that were within the lowest and highest elevational limits ever recorded for the species in field guides or in the BirdLife factsheet (BirdLife International, 2012; Hilty & Brown, 1986; Restall et al., 2006). Refining by elevation prevents us from including areas that are potentially not occupied by a species (Harris & Pimm, 2008).

Finally, we mapped the ranges of the 56 endemic and small-range bird species whose distributions were restricted to or included the Central Andes. We then looked for areas that had high concentrations of species.

### Conservation and restoration areas

We consider potentially good conservation areas those that have a high risk of landslides (as shown by our landslide susceptibility index), and high concentrations of endemic and small-range bird species (as depicted by our bird maps) in areas covered by forest. We define restoration areas as those that have these same characteristics, but lack forest cover. To visualize the results, we divided landslides into two categories based on our landslide susceptibility index (0–60 and 60–100) and divided bird concentrations into two categories (0–6 species and 7–14 species). Then, we compared these two layers to find conservation areas where high landslide risk and a high concentration of endemic and small-range birds overlapped. To identify restoration areas in the Rio Blanco reserve, we overlapped our conservation priorities with forest cover to find potential areas for restoration. We narrowed the priorities further by ordering areas according to their restoration urgency using kernel density in ArcMap 10.2 (ESRI, 1999–2010). As first priority we selected those areas very close to known landslides, and second and general priorities were further away from the landslide center.

For the Central Andes, we mapped human population density (Tatem et al., 2013) and main roads in relation to the conservation and restoration areas prioritized in our exercise. We would have liked to have mapped the main water pipe conducts but this information is not publicly available. Our aim was to evaluate the extent to which restoring the proposed areas would prevent landslides on main roads or near populated areas, thus providing an ecosystem service.

## Results

**Figure 2:**
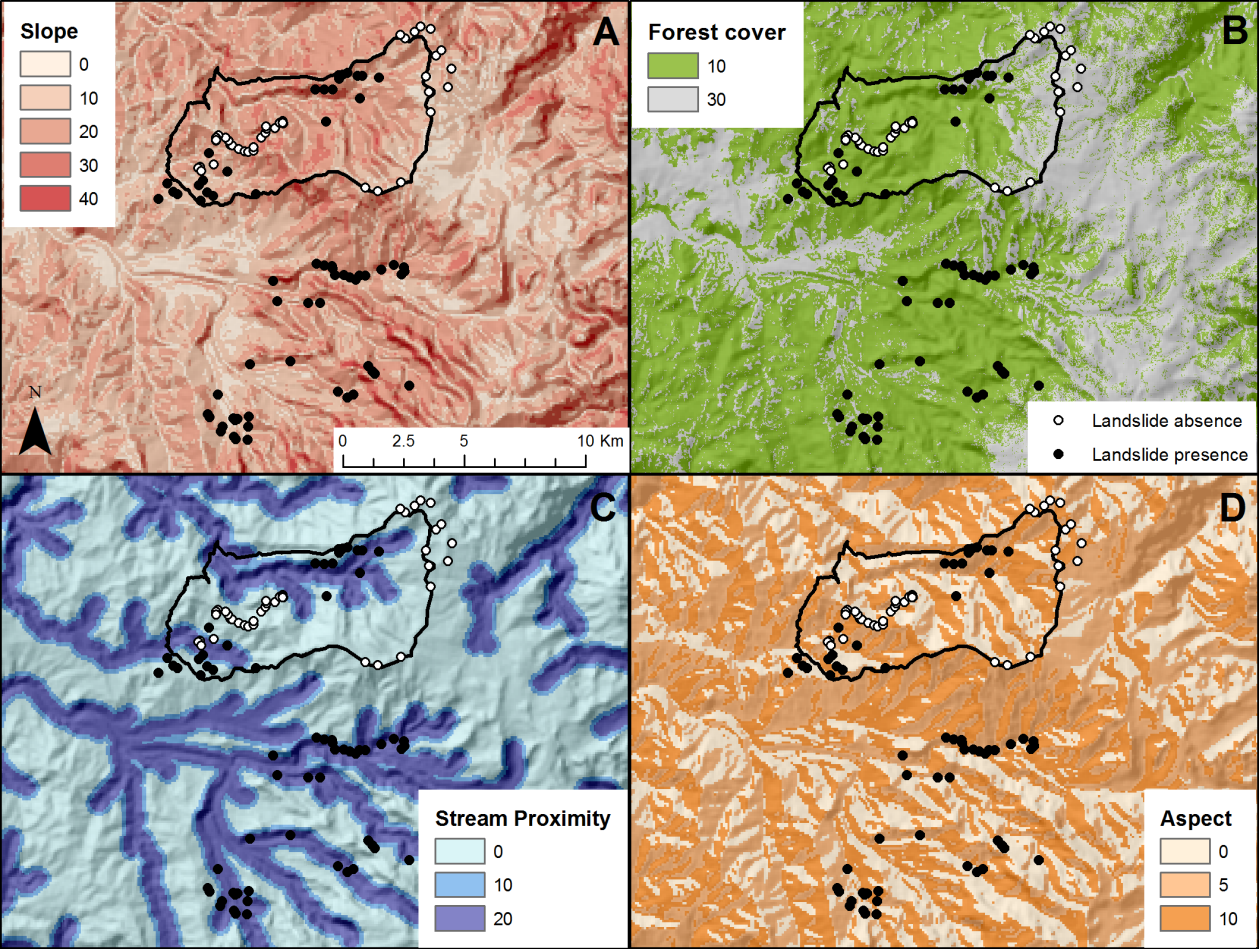
Input layers for the landslide susceptibility index in and near the Rio Blanco Reserve. All values are standardized to the 100-point scale of the landslide susceptibility index. (A) Slope derived from Digital elevation Index (Jarvis et al., 2008). (B) Forest cover derived from the Hansen et al. (2013) forest map. (C) Stream proximity derived from a stream layer. (D) Aspect derived from Digital elevation index (Jarvis et al., 2008).

In order to identify landslide susceptibility in the Central Andes of Colombia, we created an index using slope, aspect, stream proximity, and forest cover (Fig. 2). For landslide sites, the forest layer showed that 77% of the points lacked forest. As for slope, 4.5% sites were on slopes with a weighted value of 30, 50% a 20 value, and 43% a 10 value. Thirty-four percent were located near a stream (0–300 m). Twenty-eight percent were on the southern aspect, which is most vulnerable to landslides. Sites without a landslide showed a different pattern. Although for forest cover the values were similar, with 70% of the points lacking forest for the absence points, only 6.9% of the points were in the 30 slope category and 24% in the 20 slope category, less steep in general. Only 7% of the points fell within the most vulnerable stream proximity category, and 15% within the most susceptible aspect.

Our landslide susceptibility index had an overall accuracy of 55% based on the confusion matrix (Table 2). The index predicted past landslides correctly 90% of the time, but it tended to overestimate landslide presence, i.e., the commission error. In 39% of the time landslide absence was predicted accurately, i.e., omission error. Nonetheless, 90% of the time the index predicted a lack of landslides where observed points confirmed the absence. Two factors affected the accuracy of the model: (1) some landslide absence points are in paramos, naturally tree-less ecosystems (van der Hammen & Cleef, 1986); and (2) some landslide presence points were inside the forest, in areas that not visible to remote sensors, but clearly identified as dangerous by field workers.

**Table 2:** Confusion matrix for landslide susceptibility index in the Rio Blanco Reserve.

		Observed			
		Landslide	No landslide		
Predicted	Landslide	27	41	0.40	User’s accuracy
	No landslide	3	26	0.90	
		0.90	0.39	**0.55**	**Overall accuracy**
		Producer’s accuracy			

**Figure 3:**
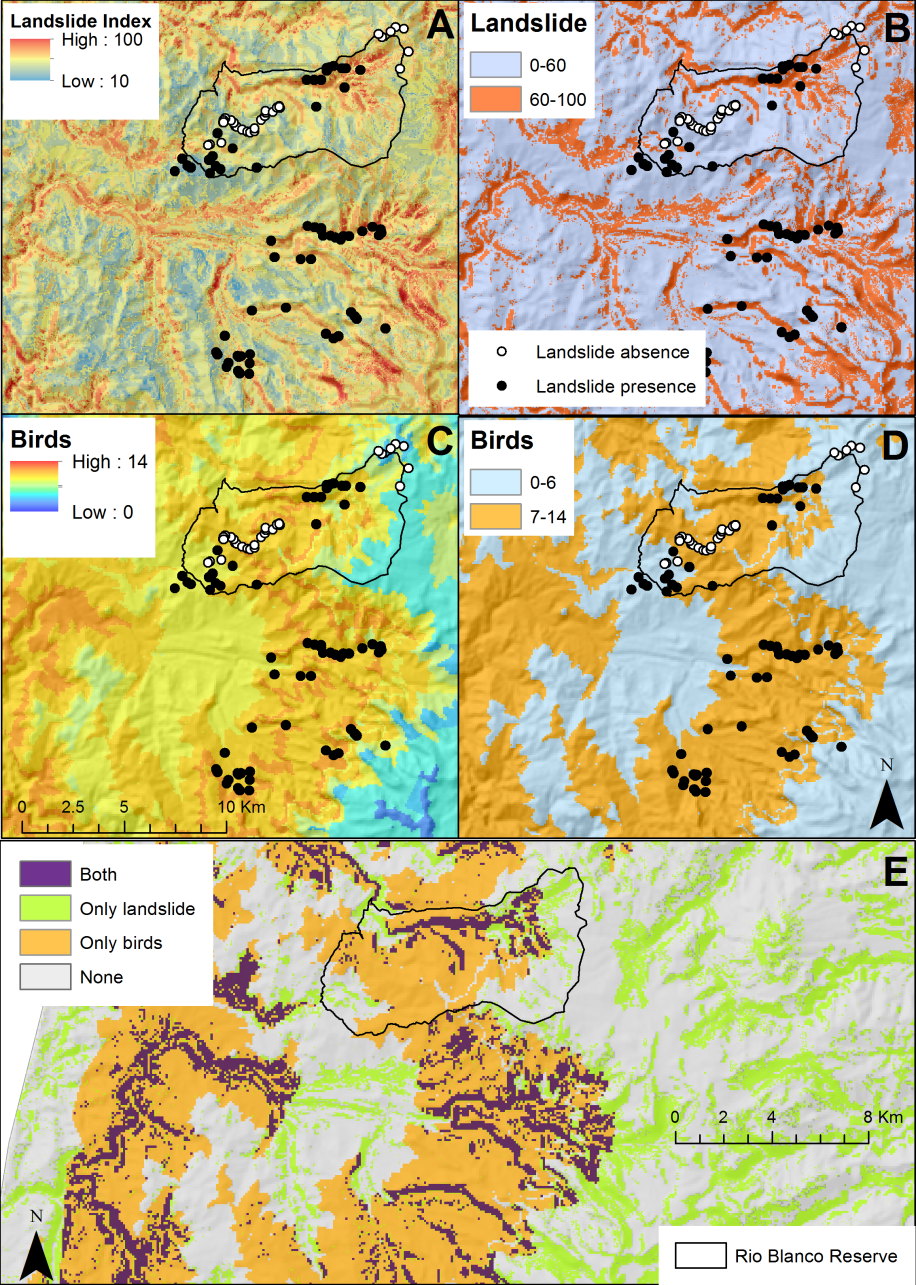
(A) Landslide susceptibility index for the Rio Blanco Reserve and its surroundings. (B) Simplified landslide susceptibility (0–60, and 60–100). (C) Concentration of endemic and threatened bird species. (D) Simplified concentration of endemic and threatened bird species (0–6, and 7–14 species). (E) Areas with high landslide susceptibility, high concentrations of endemic and threatened bird species, both (conservation priorities), or none.

To compliment landslide susceptibility, we mapped endemic and small-ranged bird concentration (Figs. 3C–3D). Maximum concentration in a given place was 14 species and it was mostly concentrated at mid-elevations, excluding paramos (above 3,000) and sub-Andean forests. Over half of the Rio Blanco Reserve fell within the 7–14 species category indicating the importance of this site for bird conservation.

**Figure 4:**
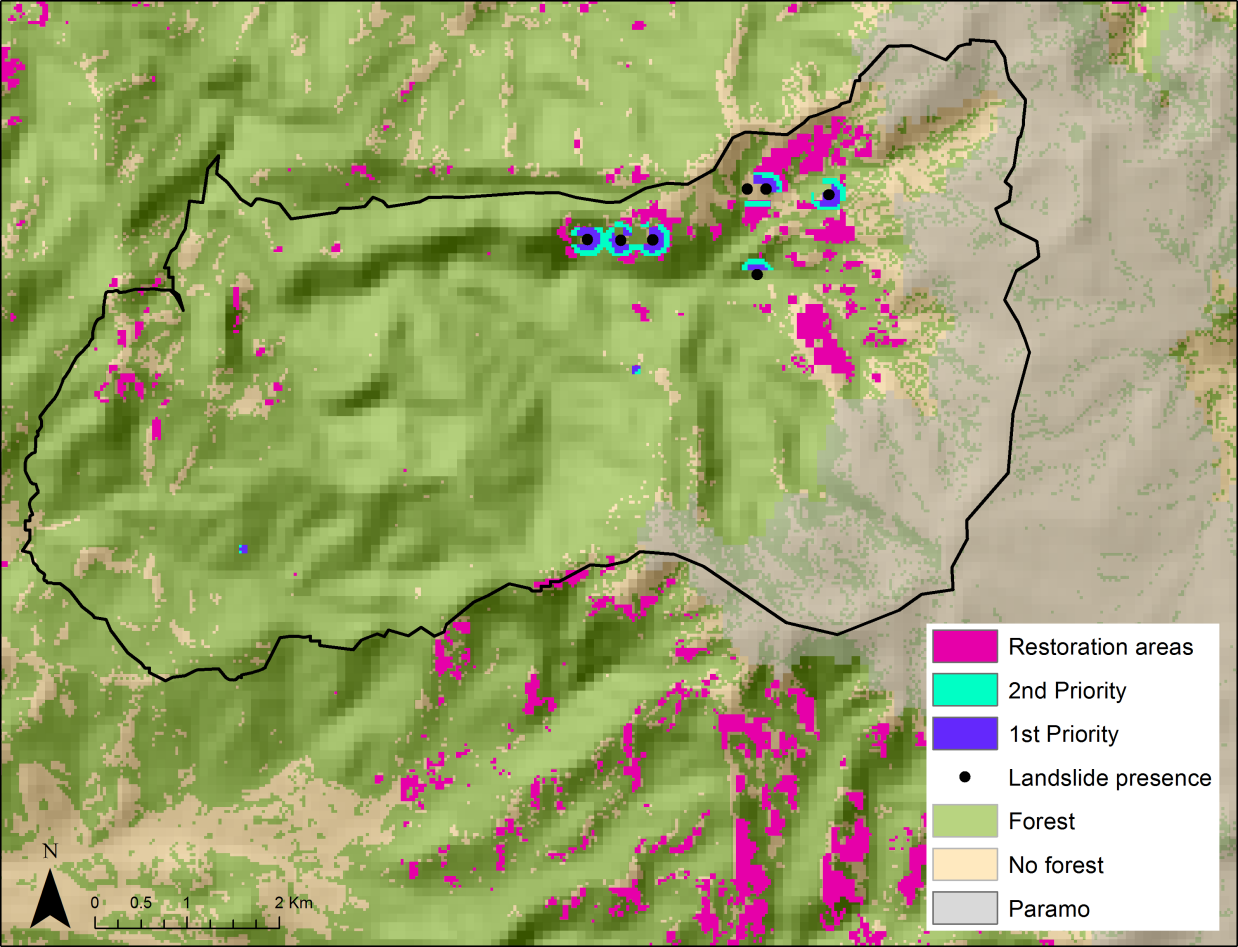
Restoration priority areas that lack forest cover and have high landslide risk and high endemic and small-range bird concentrations (first, second, and general priority) in the Rio Blanco Reserve. Paramo ecosystem (above 3500 m) shown in gray.

We simplified endemic and small-range bird concentration and the landslide susceptibility index layers (Fig. 3). Purple pixels correspond to areas that we consider appropriate for conservation due to high landslide susceptibility and endemic and small-range bird concentration. We identified 5.5 km^2^ as potential conservation areas. Some of these conservation areas were inside forest (72%), and a few were in cattle pasture or crop land (28%). We overlapped these conservation priorities with a forest cover map to further prioritize restoration areas lacking forest (Fig. 4), leaving 1.57 km^2^ as high priority for restoration. Of these, 0.21 km^2^ were first priority, 0.24 km^2^ were of secondary priority, and the remaining 1.12 km^2^ were of general priority.

**Figure 5:**
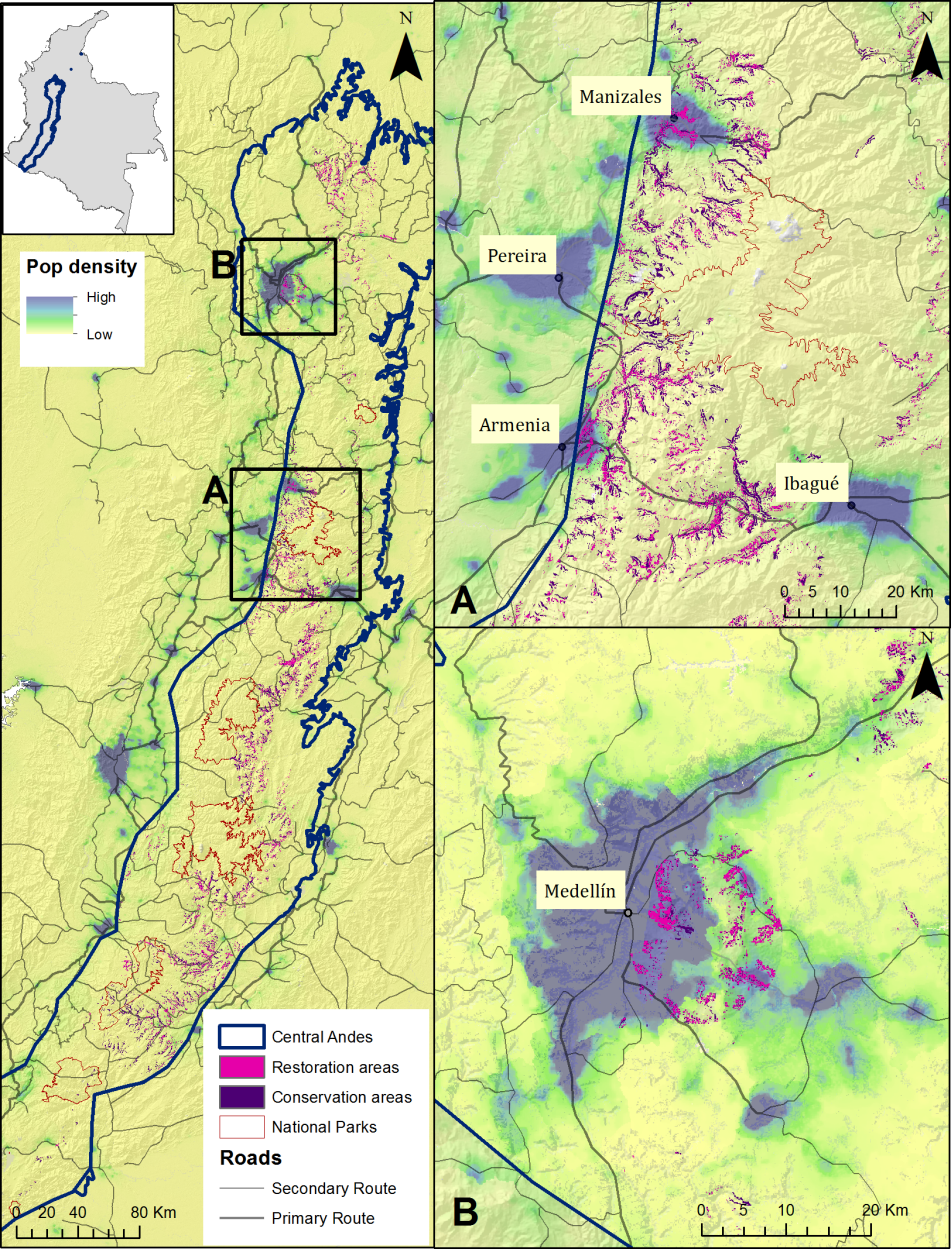
Conservation and restoration priorities, and human population density for the Central Andes. Conservation and restoration areas in the Central Andes in Colombia overlaid on layers of population density (as people per pixel, from the WorldPop dataset (Tatem et al., 2013)), and roads for the coffee-growing region (A), and the Medellin area (B).

Using the method that we developed for the Rio Blanco Reserve, we mapped conservation and restoration priorities for the Central Andes using the same criteria: landslide susceptibility, endemic and small-ranged bird concentrations, and forest cover, but added population density and roads (Fig. 5).

We identified 1980 km^2^ of potential conservation areas in the Central Andes region that present high landslide susceptibility and high concentrations of endemic and small-ranged bird species. This accounts for 27% of the total area. After overlaying these priorities on areas lacking forest, 886 km^2^ (12%) remained potential areas of forest cover restoration. To further prioritize restoration areas, we examined the location of the restoration areas in relation to roads and human population density. We consider those areas close to densely populated areas and roads, of crucial importance. Areas we highlight (Figs. 5A–5B) include major cities of Colombia like Medellin, Pereira, Manizales, Armenia, and Ibague, all with populations above 400,000.

## Discussion and Conclusions

Inspired by the November 11th landslide event near Manizales, and the current poor results of Article 111 as a conservation measure (Rudas, 2010), we set out to prioritize conservation and restoration areas where landslide prevention would be coupled with bird conservation.

We found landslides to be common on steep slopes and areas that lack forest cover. Lack of forest cover is a main contributor to landslide susceptibility, and a major triggering effect (Dai & Lee, 2002; Huggel et al., 2010; Keefer & Larsen, 2007). Steepness is often associated with landslide susceptibility (Carrara et al., 1991; Dai, Lee & Ngai, 2002). Additionally, Perotto-Baldiviezo et al. (2004) found slope and landcover to be the main determinants for landslide hazard in Honduras, in accordance with our data in Colombia.

Our landslide susceptibility index had an overall accuracy of 55%. Although not the ideal 85% accuracy proposed for landcover classification (Congalton, 1991; Nishii & Tanaka, 1999), our index predicts 90% the known landslides acting as a strong susceptibility measure and exhibiting great potential for restoration to prevent landslides.

Landslides are serious threats to human lives, social welfare, and local economies (Huggel et al., 2010). Preventing landslides is important, and the risk is highest in countries with large portions of arable land and significant resulting forest conversion, but overall high national forest cover like Colombia (Nadim et al., 2006; Farahmand & AghaKouchak, 2013).

Montane areas with high landslide susceptibility also house high concentrations of endemic and small-range bird species. We identified 5.5 km^2^ as potential conservation areas where these two conditions overlapped in the Rio Blanco Reserve. We further prioritized restoration areas by overlapping our conservation priorities with a forest cover map. A smaller area of 1.57 km^2^ is ideal for forest cover restoration, with 0.21 km^2^ being first priority, and 0.24 km^2^ second priority.

Few studies combine the protection of vulnerable species with the provision of ecosystem services as we have done for the Central Andes. Successful examples in Ecuador, Costa Rica, and New York City show watershed protection to benefit ecosystem services like potable water availability in cities (Postel & Thompson, 2005). However, the goal is often species conservation (Kattan, 1992; Orsi, Geneletti & Newton, 2011), setting new protected areas (Jenkins, Alves & Pimm, 2010), or the protection of ecosystem services alone (Chan et al., 2006; Costanza et al., 1997; Naidoo et al., 2008).

Ecosystem services are fundamental for the survivorship of all species, but the provision of these is imperiled by anthropogenic activities (Daily et al., 2000). For instance, we know that whenever human activities, such as agriculture and cattle grazing, are associated with watersheds, bank stability may decrease (Allan, 2004) and soil water retention capacity increases (Harden, 2006), causing landslides. We also know that Andean birds cannot survive in the absence of forest (O’Dea & Whittaker, 2007).

As conservation biologists, we would like to purchase and set aside the 5.5 km^2^ we identified as priority for conservation. However, we know this is not possible, especially here because land is very expensive. In light of Article 111 and recent decree 0953, Aguas de Manizales has the responsibility to expand the Rio Blanco Reserve each year. With our priority setting exercise we have downsized the priority areas from 17.76 km^2^ that are currently pasture or crops to 5.5 km^2^ (31%) which are conservation priorities, and further to 1.57 km^2^ which are restoration priorities, and 0.21 km^2^ which are the most urgent sites. Rio Blanco is one of the most popular bird watching sites in Colombia thanks to the presence of endemics like the Masked Saltator and the Bicoloured Antpitta, restoration of degraded land would expand the area for bird watching and other nature-related activities. Although restoration might take several years, Aguas de Manizales has conducted paramo restoration at higher elevations and have been successful at recovering organic matter cover by using appropriate plant species. We invite other montane municipalities in Colombia to replicate this exercise. This will contribute to guide the investment of the US$ 20 million that are available for land purchase in the country, about one third of the annual budget of the National System of Protected Areas’ (Rudas, 2010).

As a first step towards the use of this method in other montane areas, we extrapolated our priority setting exercise from the Rio Blanco Reserve to the Central Andes (Fig. 5). We identified 27% of the Central Andes as potential conservation areas, and 12% as potential restoration areas. However, 886 km^2^ is a large area to purchase and restore, so we further narrowed our priorities by mapping population density and roads. Restoring priority areas near cities would enhance ecosystem services bringing economical and social benefits to the cities (Daily et al., 2000). Landslide prevention near roads contributes to lower human deaths and operation costs. In Colombia, landslides are the main cause for road closing (INVIAS-Instituto Nacional de Vias, 2014) and have been shown to generate significant damage locally (Huggel et al., 2010). Figure 5 shows several potential restoration areas in the vicinity and within the cities of Manizales, Pereira, Armenia, Ibague, and Medellin, some of Colombia’s largest populated centers. Land near cities is probably more expensive but also in more urgent need of restoration.

We presented a simple priority setting exercise for selecting conservation and restoration areas that could be purchased following Article 111 and Decree 0953’s guidelines and enhancing biodiversity and ecosystem service conservation. We understand the limitations of applying a local index to a general region like the Central Andes and encourage the application of this index in other places using complimentary variables (soil, precipitation, volcanic influence, etc.) and updated georeferenced landslide data.

## Supplemental Information

10.7717/peerj.779/supp-1 Supplemental Information 1 Bird data used in the bird analyses This table shows the names of the 56 birds used in the analyses, as well as the minimum and maximum elevations used to refine the ranges by elevation.

10.7717/peerj.779/supp-2 Supplemental Information 2 Landslide data used as input for the landslide susceptbility model This table shows the landslide presence and absence points, and their associated values for the input layers of the landslide suceptibility model.
